# The effect of mindfulness-based childbirth education intervention on fear of childbirth: systematic review and meta-analysis

**DOI:** 10.1590/1806-9282.20240167

**Published:** 2024-08-16

**Authors:** Sevgi Dinç, Esra Erdoğan, Reyhan Aydın Doğan

**Affiliations:** 1 Karabük University, Faculty of Health Sciences, Department of Psychiatric Nursing – Karabük, Turkey.; 2 Ondokuz Mayıs University, Faculty of Health Sciences, Department of Gerontology – Samsun, Turkey.; 3 Karabük University, Faculty of Health Sciences, Department of Midwifery – Karabük, Turkey.

**Keywords:** Meta-analysis, Mindfulness, Childbirth, Fear

## Abstract

**OBJECTIVE:**

The aim of this study was to describe the effect size of mindfulness-based childbirth education on the fear of childbirth.

**METHODS:**

In this study, the meta-analysis method, one of the methods of synthesising quantitative research, was used. EBSCO, PubMed, Google Scholar, WOS, and CINAHL databases were used to determine the studies to be included in the meta-analysis. The keywords such as "mindfulness", "fear of childbirth", "mindfulness-based childbirth", "mindfulness education" and "childbirth" were searched in the international literature. Four experimental studies published between 2013 and 2022 that aimed to determine the effect of mindfulness-based childbirth education on the fear of childbirth, had a full text available and met the inclusion criteria, were included in the study.

**RESULTS:**

On the analysis of the data, mindfulness-based childbirth education was found to be effective in reducing the fear of childbirth (standard mean difference [SMD]=0.117, 95%CI: −1.049: −0.419, p<0.001, I^2^=36.98%). The results of this meta-analysis indicated that mindfulness-based education provided to pregnant women was found to be effective in reducing the fear of childbirth.

**CONCLUSION:**

Mindfulness-based childbirth education is considered to be used as an effective non-pharmacological midwifery and nursing intervention in reducing the fear of childbirth in pregnant women. This review was preregistered on PROSPERO (Ref No: CRD42022316472).

## INTRODUCTION

The fear of childbirth is defined as the fear felt before, during, and after childbirth. Evaluating childbirth as negative cognitively and approaching childbirth with anxiety and fear are also used to express fear of childbirth^1^. In a study on fear of childbirth and the related factors in pregnancy involving 203 pregnant women, it was found that pregnant women showed high levels of fear of childbirth^2^. This fear prevents women from getting pregnant and giving birth^1^. The fear of childbirth has negative effects on the pregnancy process and childbirth^3^. The hormonal changes that take place in the body due to fear of childbirth suppress contractions. This prolongs the childbirth process and requires surgical interventions for the realisation of labour^1^.

Factors that may be associated with fear of childbirth have been identified in the literature. Concerns about the health condition of the infant, attitudes and behaviours of healthcare professionals and the mother's health condition lead to an elevated fear of childbirth^4,5^. The primary concerns experienced during pregnancy are related to the health of the infant^4^. The healthcare professionals should comfort the pregnant woman with appropriate techniques and avoid negative behaviours in order to prevent the pregnant woman from having a negative experience and to have a healthier childbirth process. It is considered a necessity of the care service provided by midwives and nurses to provide properly the necessary counselling to the mother and partner during the childbirth process. These methods can reduce the risks related to the childbirth process and ensure a more successful and comfortable childbirth. Trainings about the childbirth process have been found to contribute to the reduction of negative thoughts and stress levels observed in pregnant women due to labour^4,5^. Recent studies have indicated that mindfulness-based training is used as a supplement to routine care in pregnant women. Based on this information, the aim of this study was to determine the effect of mindfulness-based childbirth education on fear of childbirth by meta-analysis method.

## METHODS

### Research model

This research is a meta-analysis study. A meta-analysis refers to an analysis done to obtain an overall result by combining the results of different studies^6^. The study protocol was registered in the database of the International Prospective Register of Systematic Reviews (PROSPERO), allowing meta-analysis studies to be recorded (ID: CRD42022316472).

### Search strategy

Before the data were collected, research questions were set in accordance with the PICOS (Participants, Intervention, Comparison, Outcomes, and Study Design) method, and a literature review was conducted based on these questions. Since the national literature lacks any studies in this field, the papers in the international literature constituted the database of the study. EBSCO, PubMed, Google Scholar, Web Of Science and CINAHL (Cumulative Index to Nursing and Allied Health Literature) online databases were searched for international articles. The keywords "mindfulness", "fear of childbirth", "mindfulness-based childbirth", "mindfulness education" and "childbirth" were used during the search.

### Inclusion and exclusion criteria

On the literature review, 18 papers related to the study were reached and the sample of the study consisted of four studies that met the inclusion criteria (Table 1). The Preferred Reporting Items for Systematic Reviews and Meta Analyses (PRISMA) model was used as a guide for reporting the study data^7^. The following criteria were used to determine which studies would be included in the meta-analysis.

**Table 1:** Characteristics of the included studies.

Authors	Study design	Participants			Mean age	Intervention period	Gestational week
		Sample (n)	Intervention group	Control group			
Veringa-Skiba et al.^15^	RCT	141	75	66	Intervention: 33.11±3.92 Control: 32.72±3.86	30 min (until the moment of childbirth)	Between 16th and 26th week
Duncan et al.^13^	RCT	29	15	14	Unspecified	18 h (2 days only)	29th week
Kuo et al.^14^	RCT	106	53	53	Intervention: 34 ± 4.0 Experiment: 33.7 ± 4.8	1 h (8 week)	Between 12th and 24th week
Byrne et al.^12^	Quasi-experimental	12	12	–	Mean: 30.1±3.7	2.5 h (throughout 8 weeks)	Between 18th and 28th week

RCT: randomised controlled trial.

1. The analysis included the studies which were published between 2013 and 2022 and aimed to determine the effect of mindfulness-based childbirth education on the fear of childbirth.
2. Randomised controlled experimental or quasi-experimental studies.
3. The experimental group consisted of pregnant women and the Wijma Birth Expectation/Experience Questionnaire (W-DEQ-A) was used in the evaluation.
4. The studies in which the effectiveness of mindfulness-based childbirth education on the experimental group was reported were included.
5. The meta-analysis included the studies that met the inclusion criteria from the studies with full text available.

### Study selection and data extraction

The coding, a data extraction process, is to remove the data eligible for the study from the complex data in the studies^8^. The data were coded in a coding form prepared in Excel format before statistical analysis. The coding form included the authors and year, study design, number of people in the experimental and control groups, mean age, intervention period, and gestational week.

### Risk of bias assessment

The quality of the selected articles was evaluated by two researchers (SD and EE) with the Quality Assessment Tool (EPHPP) checklist. The evaluation of the risk of bias in all selected articles was done by two authors (SD and EE) independently using modified Cochrane tools for assessing the risk of bias, following the criteria outlined in the Cochrane Handbook for Systematic Reviews of Interventions. The other author (RAD) checked the results. The risk of bias was classified into seven domains. The bias risk for each area was classified as "low risk," "high risk" or "uncertain risk," according to the decision criteria in the "Risk of bias" assessment tool.

### Data analysis

A comprehensive Meta-Analysis programme (CMA) (Version 3.0) was used for statistical analyses of the data, effect sizes and heterogeneity analyses. While calculating the effect size, the size was determined by Hedges G^9^, a statistic that focuses on the standardisation of the outcomes achieved and the number of samples in the study. A random-effects model was used to take into account differences between subjects, intervention methods, durations and assessment tools in the included studies. Heterogeneity analyses were made by examining Tau, I_2_, H_2_ and Q values. The heterogeneity of effect sizes was assessed using Q and I_2_ statistics. The I_2_ values indicate low (25–50%), medium (51–75%) or high (>75%) heterogeneity^10,11^. As a result of the assessment made in the study, a Q value of 4.761 (p=0.19) and an I^2^ value of 36.989% were obtained. These values indicated that there was a heterogeneous structure. Due to the heterogeneous structure, the fixed effect model was analysed, and an effect size of −0.734 (95%CI: −1.049: −0.419) was found to be statistically significant at the medium level (p=0.00). In order to render the effect size of −0.734 obtained according to the fixed-effect model insignificant (taking 0.001), Orwin's fail-safe N value was obtained as 716. This means that the study should include approximately 179 articles with statistically insignificant results for each study included in the meta-analysis in order to render the effect size insignificant. In Kendall's tau analysis, a test value of 0.34 was obtained, which indicated that there was no publication bias (p=0.367). Based on Egger's regression analysis method, the β^0^ value was obtained as −1.203, the t-value as 0.555 and the p-value as 0.317. This result indicated that there was no publication bias.

## RESULTS

When the total of the four studies included in the study was considered, the mean age of the pregnant women ranged between 30 and 33 years. The participants consisted of both multiparous and nulliparous pregnant women who were in the 12th–29th gestational week, received mindfulness-based childbirth education during pregnancy and for whom the effectiveness of the education was assessed with the Wijma Childbirth Expectancy/Experience Questionnaire (W-DEQ-A). Table 1 shows the characteristics of the participants, the intervention details, the outcome measures of the studies and additional information about the intervention in experimental and control conditions (Table 1).

**Figure 1:**
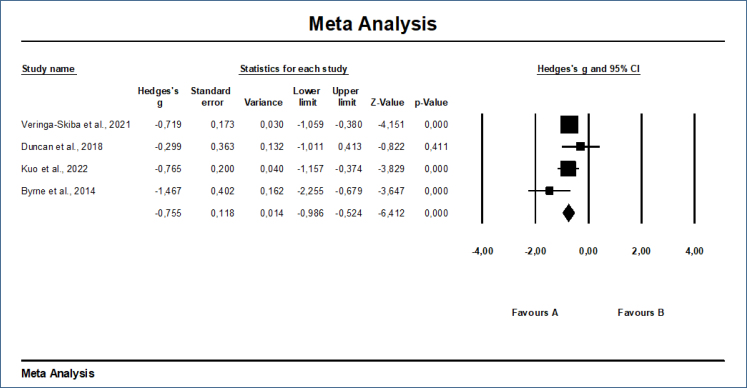
Meta-analysis outcomes.

In the study, Q statistics and I^2^ values were analysed for the heterogeneity test. As a result of the analysis, the Q value was 4.761 (p=0.19) and the I^2^ value was 37.99%. These values indicated that there was a heterogeneous structure. Due to the heterogeneous structure, the fixed-effect model was analysed, and an effect size of −0.734 (95%CI: −1.049: −0.419) was found to be statistically significant at the medium level (p=0.00). In order to render the effect size of −0.734 obtained according to the fixed-effect model insignificant (taking 0.001), Orwin's fail-safe N value was obtained as 716. Kendall's tau analysis resulted in a test value of 0.34, which indicated that there was no publication bias (p=0.367). Based on Egger's regression analysis method, the β^0^ value was obtained as −1.203, the t-value as 0.555 and the p-value as 0.317 (Figure 1 and Table 2).

**Table 2:** Homogeneity results of the meta-analysis and random-effects model (k=4) results of the meta-analysis.

Tau	Tau^2^	I^2^	H^2^	R^2^	Df	Q	p
0.038	0.194 (SE=0.085)	37.99%	-0.714		3	4.761	0.000
Random-effects model (k=4) results of the meta-analysis	Estimate	se	Z	p	CI lower bound	CI upper bound	
Intercept	-0.714	0.117	-6.086	0.000	-0.944	-0.484	

All four articles showed that awareness-based childbirth education provided to pregnant women alleviated the fear of childbirth^12–15^. The studies compared routine antenatal care with mindfulness-based childbirth education. The results of two studies showed that mindfulness-based childbirth education was more effective in alleviating the fear of childbirth compared to routine care (Byrne et al., p<0.01; Kuo et al., p<0.001), while the other two studies found that W-DEQ scores were lower than the scores obtained before the education and the difference between them was not statistically significant (Duncan et al., p=0.48; Veringa et al., p=0.045).

## DISCUSSION

Fear of childbirth is an experience with negative consequences for maternal and newborn health^16^. Fear of childbirth, experienced at mild, moderate and severe levels, can lead to complications during childbirth, difficulties in the mother–infant relationship and depression and anxiety disorders in pregnant women^1^. Birth, known as a miraculous experience in the life cycle of women, is perceived as a threat for some women and fear of childbirth appears. Here, it is highly important to transform the woman's perception of childbirth from "fear" to a positive perception. One of the methods reported to be effective in reducing fear of childbirth in recent years is mindfulness-based approaches. The mindfulness-based practices, executed from the prenatal period, focus on breathing practices, attachment with the infant and feeling emotions and the use of these practices during childbirth^16^. In this present comprehensive meta-analysis of the effectiveness of mindfulness-based childbirth education in reducing the fear of childbirth in pregnant women, it was found that mindfulness-based education provided to pregnant women was effective in reducing fear of childbirth.

Risk factors for fear of childbirth should be determined with a detailed history taken during pregnancy follow-up, and fear of childbirth in pregnant women should be assessed. Once the level of fear of childbirth has been determined, interventions such as education, counselling and childbirth support can be provided to reduce the fear of childbirth and to inform about childbirth^1^. In a study, it was reported that women's fear of childbirth reduced with the training provided by healthcare professionals^2^. A systematic review study assessing mindfulness and perinatal mental health showed that mindfulness-based programmes of 8 weeks applied to pregnant women lowered perceived stress, anxiety and depressive symptoms and the level of postpartum depression in pregnant women. It was concluded that mindfulness-based programmes elevated the levels of mindfulness and self-compassion of pregnant women^17^.

The use of a mindfulness-based model in training for childbirth preparation has a positive effect on reducing fear of childbirth and on maternal and neonatal health^16^. A randomised controlled study conducted with 63 pregnant women showed that participants in the intervention group underwent mindfulness-based cognitive behavioural therapy, while women in the control group had only routine antenatal care. In the study, it was found that the mean anxiety and depression scores of the intervention group were significantly lower than the scores of the control group^18^. A randomised controlled study conducted with 96 pregnant women showed that the intervention group attended a mindfulness-based childbirth and parenting programme, while the control group attended routine childbirth preparation education classes. The 8-week mindfulness-based programme effectively lowered the perceived stress level and depression in pregnant women and raised self-efficacy and mindfulness in childbirth^19^. In another study conducted on 60 pregnant women having their first pregnancy with 24–36 gestational weeks, the pregnant women in the intervention group were subjected to a mindfulness-based stress alleviation programme along with routine care. Immediately after the intervention and 1 month later, it was observed that there were significant reductions in anxiety symptoms of pregnant women^20^. Studies in the literature show that mindfulness-based childbirth education is effective in improving the mental health condition of pregnant women and reducing the fear of childbirth.

## CONCLUSION

Mindfulness-based education provided to pregnant women was found to be effective in reducing the fear of childbirth. It is considered that the integration of mindfulness-based education into routine pregnancy follow-ups may have positive effects on the psychological well-being of pregnant women. It is considered that reducing the fear of childbirth would increase vaginal childbirth rates and lower caesarean section rates, as well as provide a comfortable childbirth experience for pregnant women. Large-scale studies that have been meticulously designed are required to further confirm the results of this meta-analysis.

## Data Availability

Example template data collection spreadsheets are available from authors on reasonable request.
